# Dyslipidemia paradox: Analysis from the veterans exercise testing study

**DOI:** 10.1371/journal.pone.0287923

**Published:** 2023-07-19

**Authors:** Baruch Vainshelboim, Jonathan Myers

**Affiliations:** 1 Center for Tobacco Research, Division of Medical Oncology, Department of Internal Medicine, the Ohio State University, Columbus, OH, United States of America; 2 Cardiology Division, Veterans Affairs Palo Alto Health Care System and Stanford University, Palo Alto, CA, United States of America; Muhimbili University of Health and Allied Sciences School of Medicine, UNITED REPUBLIC OF TANZANIA

## Abstract

**Background:**

Dyslipidemia is a well-established cardiovascular disease (CVD) risk factor, although its association with mortality is less clear. This study aimed to assess the association between established dyslipidemia criteria [National Cholesterol Education Program (NCEP) Expert Panel on Detection, Evaluation, and Treatment of High Blood Cholesterol in Adults [Adult Treatment Panel (ATP) III] and all-cause mortality in men.

**Methods:**

Prospective cohort study of 1,479 men aged 59.7±10.7 years was conducted between 1987 and 2012. At baseline, dyslipidemia markers of total cholesterol (TC), low-density lipoprotein cholesterol (LDL-C) and high-density lipoprotein cholesterol (HDL-C) were assessed as an exposure. Cox proportional hazard models were analyzed adjusting for conventional health risk factors using all-cause mortality as an outcome.

**Results:**

Mean and standard deviations of TC, LDL-C and HDL-C were 199.5±45.2, 149.4±47.4 and 44.3±12.2 mg/dL, respectively. During 8.9±4.5 years follow-up, 284 participants died. Compared to TC <200 mg/dL, levels of 200–239 mg/dL and ≥240 mg/dL were associated with 13% [hazard ratio (HR) = 0.87, 95% confidence intervals (CI) (0.66–1.1)] and 37% [HR = 0.63, 95% CI (0.44–0.92)] lower risks of mortality (p trend = 0.048), respectively. Compared to LDL-C <130 mg/dL, levels of 130–189 mg/dL and ≥190 mg/dL were associated with 26% [HR = 0.74, 95% CI (0.57–0.97)] and 32% [HR = 0.68, 95% CI (0.48–0.98)] lower risks of mortality (p trend = 0.044), respectively. Mean survival time was 0.9 to 1.9 years longer with higher TC and LDL-C categories (both p = 0.001). HDL-C was not associated with mortality.

**Conclusion:**

In reference to established dyslipidemia criteria, this study showed that higher TC and LDL-C were independently and paradoxically associated with lower risk of all-cause mortality and longer survival time in men. Along with previous reports, these novel findings support a rigorous reevaluation of evidence on dyslipidemia and health risks. Systematic review and meta-analysis are warranted for evidence-based recommendations on dyslipidemia for primary and secondary prevention of CVD.

## Introduction

Dyslipidemia is a well-established risk factor for atherosclerotic cardiovascular disease (CVD) [1–3]. The most widely adopted definition of dyslipidemia is based on the Third Report of The National Cholesterol Education Program (NCEP) Expert Panel on Detection, Evaluation, and Treatment of High Blood Cholesterol in Adults [Adult Treatment Panel (ATP) III] [3]. According to the NCEP panel, total cholesterol (TC) ≥240 mg/dL and low-density lipoprotein-cholesterol (LDL-C) ≥190 mg/dL are considered to be high and very high levels, respectively, and are indications for lipid lowering therapy [3].

From a pathophysiological perspective, it is believed that lipoproteins <70 nm in diameter such as LDL-C can cross the endothelial barrier in the presence of endothelial dysfunction, and can become trapped in the arterial wall, provoking a complex process that leads to lipid deposition and the initiation of an atheroma [2]. It has been assumed that higher plasma cholesterol concentrations lead to additional particles being retained and faster lipid accumulation in the arterial wall, resulting in more rapid growth and progression of atherosclerotic plaques in the arteries [2]. Eventually, the increase in the atherosclerotic plaque burden reaches a critical point at which disruption of a plaque and the formation of an overlying thrombus can result in acute blood flow obstruction, unstable angina, myocardial infarction or death [2]. This pathophysiological mechanism provides a rationale for maintaining low levels of TC and LDL-C throughout life in order to slow the progression of atherosclerosis for primary and secondary prevention of CVD [1, 2]. Despite this sound notion, a body of literature suggests that TC and LDL-C are less predictive of CVD risk, whereas small and oxidized LDL-C particles, very low density lipoprotein cholesterol (VLDL-C) and apolipoprotein type B (ApoB), have a mechanistic contribution to atherosclerotic plaques and are more accurate in CVD prognostication [1, 2, 4–6]. In addition, there is an emerging body of evidence suggesting contradictory results with respect to the health hazards of high cholesterol levels [7–19]. For instance, a large prospective cohort of 12.8 million adults from Korea reported an 8% lower all-cause mortality risk for each 39 mg/dL increase in TC. This study also showed an increased mortality risk ranging from 10% to 240% for those with TC <200 mg/dL [9]. The Honolulu Heart Program study of 3,572 Japanese/American men (aged 71–93 years) with a 20 year follow up showed that compared to men in the lowest quartile of TC (81–167 mg/dL), men in the second (168–188 mg/dL), third (188–210 mg/dL), and fourth (210–383 mg/dL) quartiles had 27%, 38% and 31% lower all-cause mortality risks, respectively [11]. The Copenhagen General Population Study followed 108,243 men and women from Denmark for a median of 9.4 years [7]. Although the study showed a 15% increased mortality risk among individuals with LDL-C levels >189 compared to individuals in the range of 132–154 mg/dL, those who had lower LDL-C levels of 113–131, 93–112, 70–92 and <70 mg/dL exhibited 7%, 8%, 18% and 25% higher all-cause mortality risks, respectively [7]. In addition, the latter study reported that LDL-C levels between 155–189 mg/dL (average 170 mg/dL), levels that are traditionally considered hazardous even for healthy individuals, were not associated with mortality [7]. Surprisingly and paradoxically, this study also found that higher LDL-C levels in a continuous model were associated with lower risks of fatal stroke and total cancer incidence [7]. Results from the China Health and Retirement Longitudinal Study of 4,981 men and 5,529 women demonstrated no association between LDL-C and mortality among women but significantly reduced risk among men with higher LDL-C [10]. Compared to men in the first quintile (LDL-C ≤83.9 mg/dL), men in the second, third, fourth and fifth quantiles had 27%, 36%, 48% and 49% lower risks of all-cause mortality, respectively [10]. Finally, a systematic review of 19 prospective cohort studies among individuals ≥60 years found that higher LDL-C was inversely associated with all-cause mortality in 14 of these studies, whereas the other 5 did not show any association with mortality [18]. Given the variation in these studies with respect to risk associations between TC, LDL-C levels and mortality [7–19], there is a need to explore the specific risk association between common clinical criteria for dyslipidemia and mortality outcomes. This knowledge has important practical implications for risk stratification criteria based on clinical guidelines for dyslipidemia [3]. Therefore, the current study aimed to assess the prospective association between dyslipidemia markers using established clinical criteria (NCEP-ATP III) and all-cause mortality in men.

## Methods

### Study design and population

**Fig 1:**
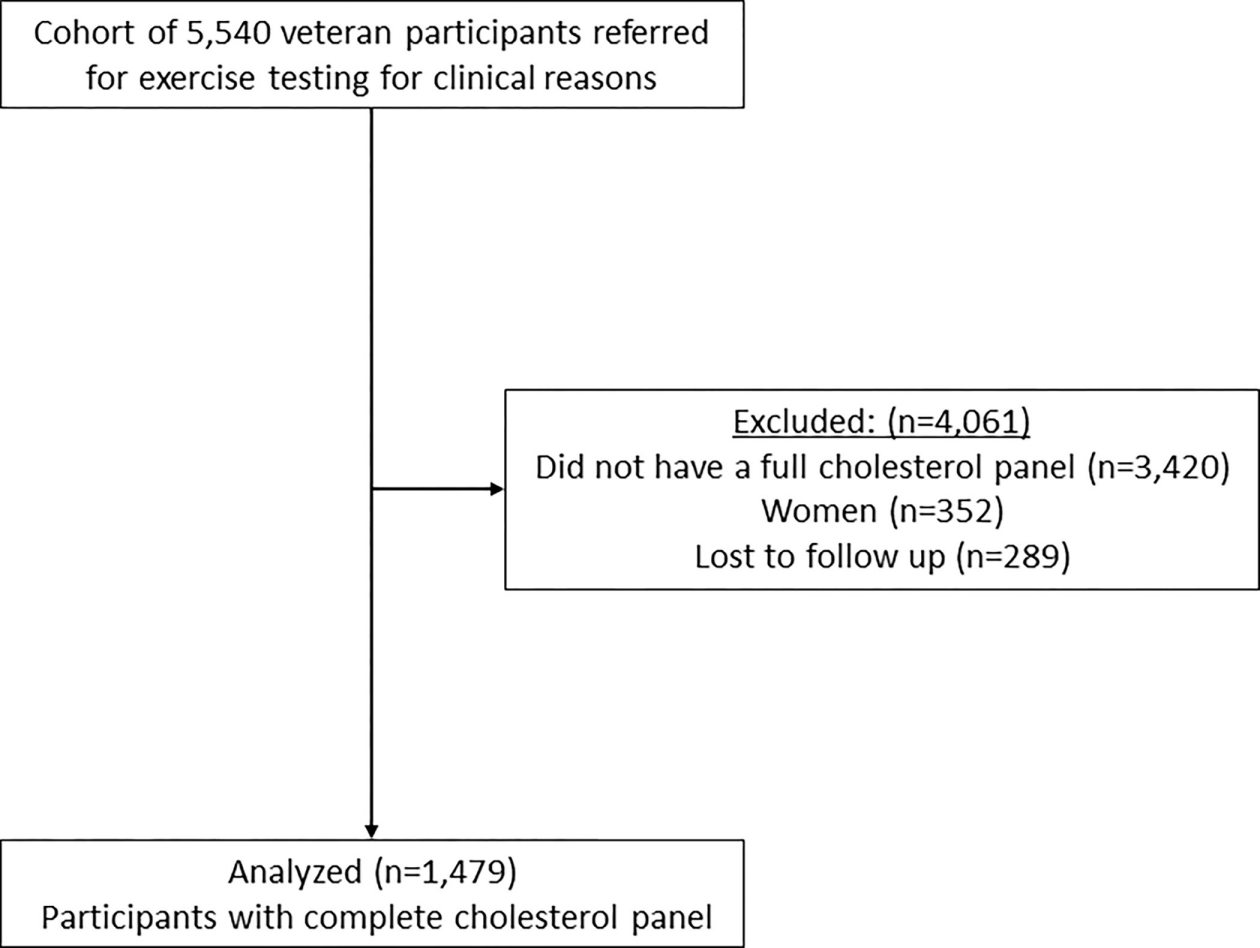
Flowchart of study design.

The present analysis was carried out under the framework of the Veterans Exercise Testing Study (VETS), which has been previously described [20]. In brief, the VETS cohort is an ongoing, prospective evaluation of primarily male (96%) Veterans referred for exercise testing for clinical reasons, designed to address the association between exercise test, clinical, lifestyle factors and health outcomes. The sample generally included participants with cardiometabolic risk factors, signs or symptoms suggestive of cardiovascular disease, or known cardiometabolic disease. The study was approved by the Institutional Review Board at Stanford University, CA, USA. All participants who underwent a treadmill exercise test at the Veterans Affairs Palo Alto Health Care System between 1987 and 2012 were considered for inclusion, and written informed consent was obtained prior each test. Clinical information on diagnoses, risk factors and health-related behaviors (smoking, physical activity, cholesterol levels) were collected at the time of the exercise test using the Veterans Affairs Computerized Patient Record System (CPRS) and self-reported health history. Of 5,540 participants who completed the baseline evaluation, 4,061 were excluded from the analysis [did not have a full cholesterol panel (n = 3,420), women (n = 352) and lost to follow up (n = 289)]. A total of 1,479 men with a complete cholesterol panel at baseline were included in the current study (**Fig 1**).

### Exposure-cholesterol assessment and dyslipidemia definition

Using the Veterans Affairs CPRS medical records, serum cholesterol levels were obtained from each participant during the day of the exercise test, or recent blood work performed within three-months of the exercise test. Blood sample collection and specimen analysis was conducted according to established guidelines of the lipid standardization laboratory of the US Centers for Disease Control and Prevention [21]. Blood samples were drawn from an antecubital vein by medically trained staff after an overnight fast of 8–12 hours. Samples were centrifuged and stored at –80°C until cholesterol concentration levels were measured using the enzymatic colorimetric test [21]. Dyslipidemia was defined according to the NCEP-ATP III guidelines [3]. Participants who met one or more of the dyslipidemia criteria for TC ≥240 mg/dL, LDL-C ≥190 mg/dL and HDL-C<40 mg/dL were determined to have dyslipidemia [3].

### Outcome-mortality ascertainment

The Veterans Affairs CPRS was used for capturing mortality outcomes; all-cause mortality was the primary outcome. Previous reports have demonstrated that the Veterans Affairs death records are relatively complete compared to those from other sources, such as the Social Security Administration [22]. The Veterans Affairs records also have excellent agreement (Cohen’s kappa coefficient (ĸ) = 0.82–0.91) with state death records [23]. Vital status for each participant was ascertained as of December 31, 2012.

### Statistical analysis

Demographic and clinical data of the participants are presented as mean ± standard deviation. Categorical variables are presented in percentages. Comparisons between participants with and without dyslipidemia were performed using independent t-tests for continuous variables and chi-square tests for categorical variables. Multivariable Cox proportional hazard models were used to assess the association between dyslipidemia status, TC, LDL-C, HDL-C and all-cause mortality.

The risk models were adjusted for established health risk factors utilized in previous studies [7–17]. These included age, obesity status (body mass index ≥30), hypertension, diabetes, smoking status (never, former, current), family history of cardiovascular disease, alcohol abuse, use of anti-hypertensive medications, use of statin medications and presence of any cardiovascular disease. Survival analysis of TC and LDL-C categories was conducted using Kaplan–Meier curves and log-rank tests. To test the proportional hazards assumption, scaled Schoenfeld residuals and graphical evaluation of the Kaplan–Meier curves were performed with no major violation evident.

Sensitivity analyses were performed for potential bias and confounding of the observed association between TC and LDL-C and mortality outcomes. E-Value was calculated to measure an association’s robustness for potential uncontrolled confounders. The E-value is a continuous variable, where 1 is the lowest possible value indicating no unmeasured confounding is needed to explain away the observed association between exposure and outcome. Conversely, the higher the E-value, the stronger the confounding associations that must be explained in the relationship between exposure and outcome [24]. Stratified analyses by age categories, cardiovascular disease status, presence of diabetes and the use of statin medications were also conducted. To address the potential for reverse causality, analysis was performed excluding participants with less than five years follow-up. For selection bias, Little’s MCAR test was conducted to assess potential patterns in the missing data. The test showed that missing data were random (chi-square = 6.9, p = 0.23) and data were analyzed using the complete case method [25]. Data reporting and presentation followed the “Strengthening the Reporting of Observational Studies in Epidemiology” (STROBE) guidelines [26]. Data management and statistical analyses were conducted using IBM SPSS Statistics Software version 23.0 (IBM, Armonk, NY, USA). The significance level was set at p<0.05.

## Results

The analytical sample included 1,479 men aged 59.7±10.7 years. Of the sample, 779 (52%) had dyslipidemia by meeting one or more of the established NCEP-ATP III criteria [3]. Approximately 23% of the cohort had coronary artery disease, 21% had type two diabetes, 60% had hypertension and 40% were obese. Serum TC, LDL-C and HDL-C levels were 199.5±45.2 mg/dL, 149.4±47.4 mg/dL and 44.3±12.2 mg/dL, respectively. Demographic and clinical characteristics of the participants are presented in **Table 1**. Participants with dyslipidemia had higher body mass index levels and higher prevalence of obesity and diabetes compared to participants without dyslipidemia **(Table 1).**

**Table 1:** Baseline demographic and clinical characteristics of the cohort.

	Entire Cohort (n = 1,479)	Present with Dyslipidemia (n = 779)	Present without Dyslipidemia (n = 700)	P Value
Age (yr)	59.7±10.7	58.4±10.4	61.3±10.9	<0.001
BMI (kg/m^2^)	29.3±5.2	30.1±5.1	28.4±5.2	<0.001
Obesity (BMI≥30 kg/m^2^)	40%	47.5%	31.6%	<0.001
*Smoking Status*				
Never	28.7%	27.7%	29.9%	
Former	46.8%	45.4%	48.3%	0.084
Current	24.5%	26.8%	21.9%	
Smoking (pack/years)	34.2±30.7	34.3±28.2	34.1±33.4	0.906
Hypertension	59.9%	61.4%	58.3%	0.228
Cardiovascular Disease	23.2%	23.4%	23%	0.869
Diabetes	21.1%	24%	17.9%	0.040
Total Cholesterol (mg/dL)	199.5±45.2	211±53	186.7±29.8	<0.001
LDL-C (mg/dL)	149.4±47.4	166.8±52	129.9±32.2	<0.001
HDL-C (mg/dL)	44.3±12.2	38.1±9.9	51.2±10.9	<0.001
Anti-Hypertensive Drugs	16.2%	16.7%	15.7%	0.612
Statins Drugs	23.9%	21.7%	26.4%	0.033
Physically Inactive	47.2%	45.4%	49.3%	0.144
Cardiorespiratory Fitness (METs)	8.4±3.1	8.3±3	8.5±3.2	0.210
Deceased (n/%)	284 (19.2)	151 (19.4)	133 (19)	0.852

Data presented as means ± standard deviation for continuous variables and % or number/% for categorical variables. BMI; body mass index, HDL-C; high density lipoprotein-cholesterol, LDL-C; low density lipoprotein-cholesterol, METs; metabolic equivalents, TC; total cholesterol.

**Fig 2:**
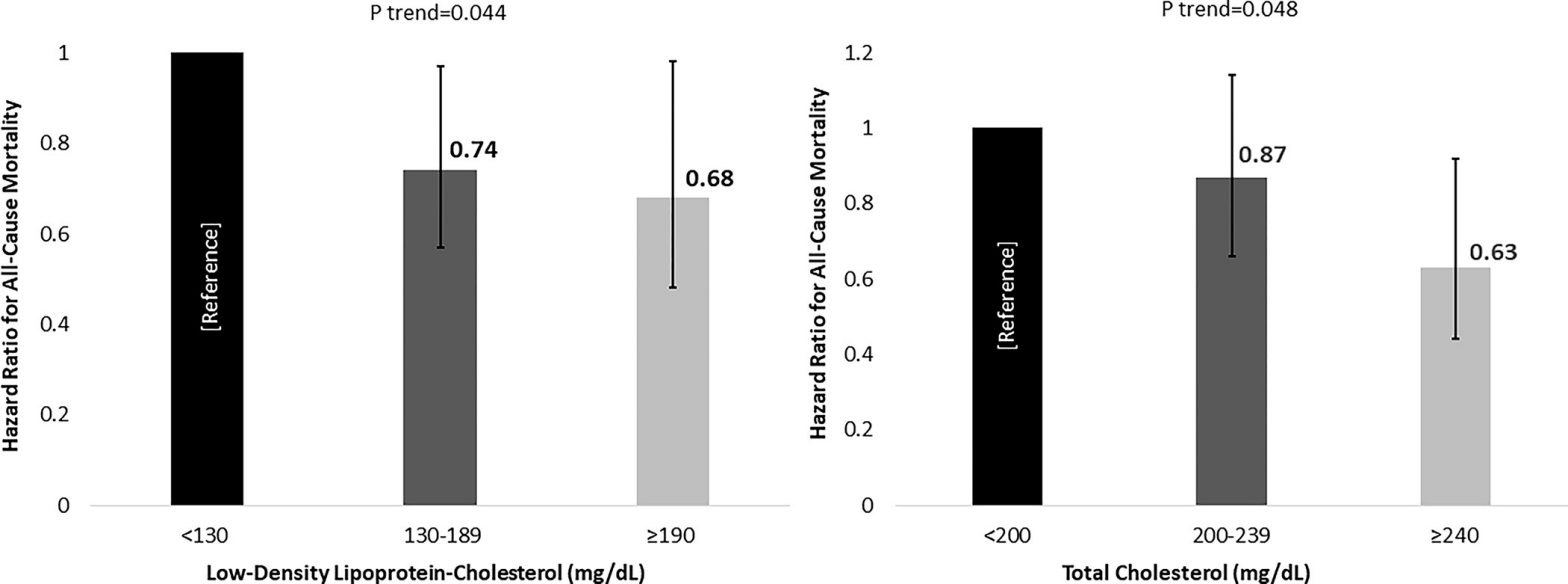
Hazard ratios in categorical analysis of total cholesterol and low-density lipoprotein cholesterol in men.

**Fig 3:**
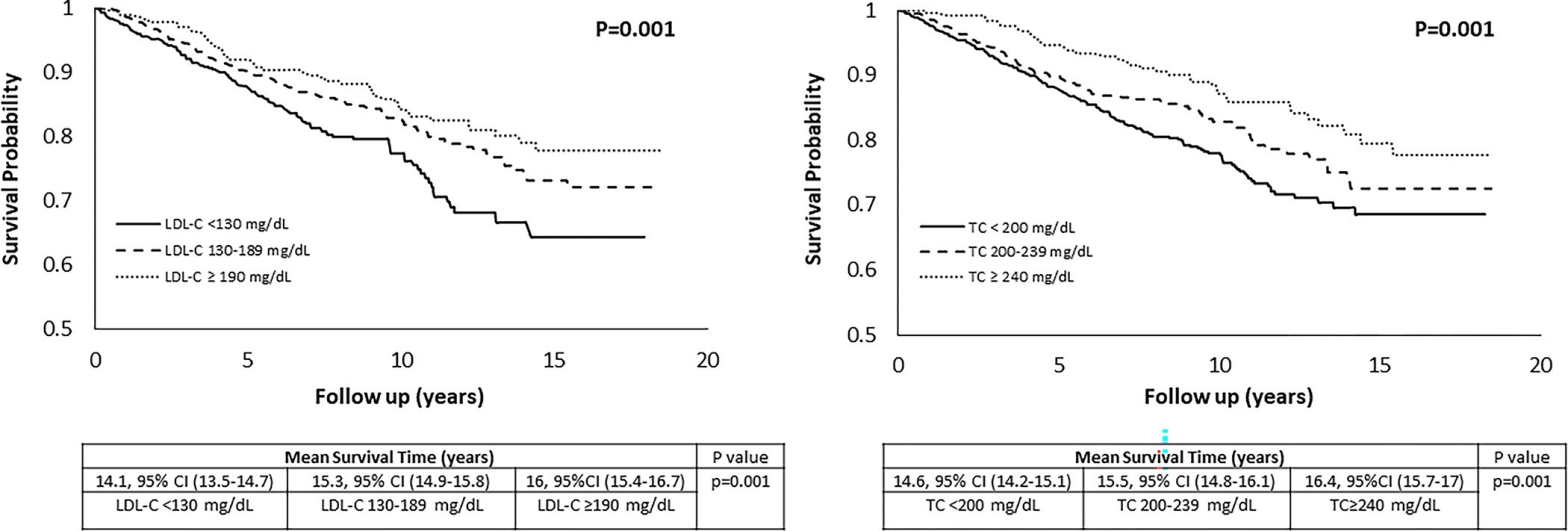
Survival analysis of total cholesterol and low-density lipoprotein cholesterol in men. LDL-C; low density lipoprotein-cholesterol, TC; total cholesterol.

During 8.9±4.5 years follow-up, 284 participants died from all-causes. In the total cohort, dyslipidemia was not associated with mortality (**Tables 1 and 2**). In the categorical risk models, compared to TC <200 mg/dL, levels of 200–239 mg/dL and ≥240 mg/dL were associated with 13% and 37% lower risks of mortality (p trend = 0.048), respectively. The corresponding hazard ratios and 95% confidence intervals were [0.87, 95% CI (0.66–1.1)] and [0.63, 95% CI (0.44–0.92)]. Compared to LDL-C <130 mg/dL, levels of 130–189 mg/dL and ≥190 mg/dL were associated with 26% and 32% lower risks of mortality (p trend = 0.044), respectively **(Fig 2)**. The corresponding hazard ratios and 95% confidence intervals were [0.74, 95% CI (0.57–0.97)] and [0.68, 95% CI (0.48–0.98)]. Mean survival time was 0.9 to 1.9 years longer among individuals at higher TC and LDL-C categories (both p = 0.001) **(Fig 3)**. In the continuous risk models, each 10 mg/dL increase in TC and LDL-C levels were associated with 10% (p = 0.001) and 9% (p = 0.01) reductions in mortality risk, respectively. HDL-C was not associated with mortality (**Table 2**).

**Table 2:** Continuous and categorical hazard models of cholesterol levels in the total cohort of men (n = 1,479).

Dyslipidemia Variables	Hazard Ratio 95% (Confidence Intervals)	P value	Per-10 mg/dL increase	P value
Dyslipidemia [meeting one or more of the criteria (TC≥240, and/or LDL-C>190, and/or HDL-C<40 mg/dL)]	1.05 (0.82–1.33)	0.718	N/A	N/A
Total Cholesterol (mg/dL) ≥240 vs <240	*0.67 (0.47–0.95)	0.026	*0.90 (0.88–0.98)	0.001
Low Density Lipoprotein-Cholesterol (mg/dL) ≥190 vs <190	0.82 (0.59–1.13)	0.231	*0.91 (0.9–0.99)	0.01
High Density Lipoprotein-Cholesterol (mg/dL) <40 vs ≥40	1.18 (0.93–1.51)	0.176	1 (0.99–1.01)	0.962

* Statistically significant. The risk models were adjusted for age, obesity, hypertension, diabetes, smoking status (never, former, current), family history of cardiovascular disease, alcohol abuse, usage of anti-hypertensive drugs, usage of statins and presence of any cardiovascular disease. HDL-C; high density lipoprotein-cholesterol, LDL-C; low density lipoprotein-cholesterol, TC; total cholesterol.

Sensitivity analyses confirmed low risk of confounding and bias, and generally showed similar results with respect to the risk association between dyslipidemia markers and mortality **(Table 3).** Participants younger than 60 years who exhibited dyslipidemia had 43% to 66% reduced risks of mortality compared to individuals with lower cholesterol levels. Meeting dyslipidemia criteria was not associated with mortality in individuals with CVD, diabetes or those on statin medications (**Table 3).** In a sub-cohort of participants, after exclusion of those with less than five years follow up, high TC and LDL-C levels were not associated with mortality. In contrast, low HDL-C (<40 mg/dL) was associated with 54% increased risk of mortality. E-value analysis of contentious risk models were 1.11, 95%CI (1.02–1.14) and 1.10, 95%CI (1.01–1.11) for TC and LDL-C, respectively, suggesting negligible unmeasured confounding.

**Table 3:** Sensitivity analyses of cholesterol levels and all-cause mortality in men.

	Hazard Ratio 95% (Confidence Intervals) and p values									
Dyslipidemia Variables	Age Categories (years)		Cardiovascular Disease		Diabetes		On Statins Medication		Sub-Cohort after Exclusion those with less than five years follow-up	E-Value Analysis per 10 dL/mg increase [Point estimate, 95% (Confidence Intervals)]
	<60 (n = 768)	≥60 (n = 711)	Present (n = 343)	Absent (n = 1,136)	Present (n = 312)	Absent (n = 1,167)	Yes (n = 354)	No (n = 1,125)	(n = 1,166)	
Dyslipidemia [meeting one or more of the criteria (TC≥240, and/or LDL-C≥190, and/or HDL-C<40 mg/dL)	*0.57 (0.36–0.91) P = 0.019	1.28 (0.97–1.71) P = 0.086	1.02 (0.69–1.5) P = 0.921	1.11 (0.81–1.5) P = 0.520	1.01 (0.6–1.7) P = 0.962	1.1 (0.81–1.4) P = 0.645	1.14 (0.5–2.5) P = 0.752	1.03 (0.8–1.33) P = 0.796	*1.44 (1.01–2.1) P = 0.042	
Total Cholesterol (mg/dL) ≥240 vs <240	*0.34 (0.16–0.72) P = 0.005	0.87 (0.58–1.32) P = 0.531	0.8 (0.44–1.45) P = 0.455	*0.62 (0.4–0.97) P = 0.036	0.66 (0.29–1.5) P = 0.324	*0.67 (0.45–0.99) P = 0.047	0.49 (0.1–3.8) P = 0.497	*0.68 (0.47–0.97) P = 0.035	0.79 (0.5–1.4) P = 0.307	1.11 (1.02–1.14)
Low Density Lipoprotein-Cholesterol (mg/dL) ≥190 vs <190	*0.48 (0.26–0.89) P = 0.019	1.07 (0.73–1.57) P = 0.717	1.03 (0.61–1.76) P = 0.902	0.74 (0.49–1.12) P = 0.154	1.12 (0.58–2.2) P = 0.732	0.74 (0.51–1.08) P = 0.117	1.7 (0.47–6.2) P = 0.418	0.78 (0.56–1.1) P = 0.163	0.8 (0.5–1.25) P = 0.333	1.10 (1.01–1.11)
High Density Lipoprotein-Cholesterol (mg/dL) <40 vs ≥40	0.83 (0.52–1.34) P = 0.443	1.33 (1–1.77) P = 0.054	1.12 (0.77–1.64) P = 0.562	1.27 (0.93–1.8) P = 0.139	1.1 (0.6–1.8) P = 0.725	1.22 (0.92–1.6) P = 0.167	1.44 (0.64–3.2) P = 0.372	1.15 (0.89–1.5) P = 0.296	*1.54 (1.1–2.2) P = 0.014	

* Statistically significant. The risk models were adjusted for age, obesity, hypertension, diabetes, smoking status (never, former, current), family history of cardiovascular disease, alcohol abuse, usage of anti-hypertensive drugs, usage of statins and presence of any cardiovascular disease. HDL-C; high density lipoprotein-cholesterol, LDL-C; low density lipoprotein-cholesterol, TC; total cholesterol.

## Discussion

The current study demonstrated that dyslipidemia defined by NCEP-ATP III guidelines [3] was not associated with higher all-cause mortality risk in men. Paradoxically and independent of other established health related risk factors, utilizing clinically established criteria for dyslipidemia [3], higher TC and LDL-C categories were associated with lower risk of mortality and longer survival time (**Tables 2 and 3, Figs 2 and 3)**. The results were strengthened by continuous risk models and sensitivity analyses, showing 9%-10% lower risks of mortality per 10 mg/dL increase in TC and LDL-C levels **(Table 2)**, and a low risk of bias and confounding (**Table 3).** While these observations do not indicate causality and require further investigation, they are in alignment with a recent body of literature [7–19] suggesting that higher TC and LDL-C levels are not associated with higher mortality risk in variety populations. Rigorous meta-analyses of the existing evidence as well as reevaluation of dyslipidemia guidelines to inform treatment are needed to ensure evidence-based management of dyslipidemia.

Although these findings are counter to conventional dogma, they are consistent with other recent reports generally showing a lack or inverse association between cholesterol levels and mortality outcomes [7–19]. The current results extend these reports by providing novel and clinically relevant data with respect dyslipidemia markers and mortality risk in men. Using established dyslipidemia criteria (NCEP-ATP III) [3], the current study is the first to our knowledge to demonstrate a paradoxically lower mortality risk and longer survival time in men who met these criteria for dyslipidemia. To overcome potential borderline cholesterol thresholds, the study utilized the highest thresholds of TC and LDL-C to define dyslipidemia [3]. Surprisingly, the results showed that the highest TC (≥240 mg/dL) and LDL-C (≥190 mg/dL) categories were associated with lower risks of all-cause mortality and extended survival time compared to lower TC and LDL-C categories **(Tables 2 and 3, Figs 2 and 3)**.

Analysis of the sub-cohort after excluding participants who had less than five years of follow-up showed an increased mortality risk in those who met the low HDL-C (<40 mg/dL) criteria only. Both TC and LDL-C were not associated with increased mortality in this sub-cohort. Thus, the increased risk of combined dyslipidemia criteria is due to low HDL-C only (Table 3). Stratified analyses by age, disease and statin medications status confirmed that higher cholesterol did not increase the risk of mortality, whereas the E-value analysis indicated low confounding risk, supporting a true association between exposure and outcome (**Table 3**). Taken together, consistent with other reports [7–19], the present study demonstrated that higher TC and LDL-C that clinically meet dyslipidemia criteria [3] were associated with lower all-cause mortality risk, warranting further exploration of this issue.

Although the exact mechanism why higher cholesterol levels might provide protective benefits against mortality in particular populations is not fully understood, there is biological plausibility which could possibly explain some of the findings. Cholesterol has many essential physiological roles both at the cellular and organism levels, whereas inadequate levels in some cases may lead to dysregulation of homeostasis and promote illness. Cholesterol is a structural material that comprises about 20% of all cells’ plasma and 60–80% of all cell membranes. At the organism level, cholesterol is a precursor for all steroid hormones, including gluco- and mineralo-corticoids, sex hormones and vitamin D, hormones that regulate carbohydrate metabolism, sodium balance and reproductive and bone homeostasis. Cholesterol is also a precursor for bile acids, an important mechanism for intestinal absorption of dietary lipids and energy and glucose regulation [27]. Low TC and LDL-C levels have the potential to underlie abnormal function at the cellular and systemic levels, while sufficient levels could provide protective benefits against disease development and death. This was partially supported in a meta-regression analysis of 15 randomized controlled trials including 51,797 statin-allocated patients and 45,043 control patients followed for 4.4 ± 1.4 years [28]. This study showed that in both arms, lower LDL-C levels were associated with higher incidence of total cancer [28], a complex group of diseases that remains the second leading cause of death in the US and worldwide [29, 30]. An analysis of nine cohort studies including more than 140,000 individuals found that low TC was associated with increased risk for incidence and mortality of cancer. The association persisted even after exclusion of cancer cases appearing during the first 4 years [31]. It is speculated that lower levels of cholesterol could possibly result in a higher cancer morbidity and mortality. This was partially supported in a study of patients admitted to a hospital for acute coronary syndrome. Low LDL-C (<105 mg/dL) at admission (average 79±19 mg/dL) compared to relatively high (≥105 mg/dL) (average 144±38 mg/dL) was associated with a two-fold higher risk of all-cause mortality and reduced 3-year survival rates (7.1% vs 14.8%, respectively) [13]. Additionally, evidence suggests that small, oxidized LDL-C particles, VLDL-C and ApoB are mechanistically involved in the initiation and progression of atherosclerotic plaques, suggesting that advanced lipid profiles including LDL-C sub-particles may be more informative for risk assessment [1, 2, 4–6]. Mechanistic and clinical studies in humans are needed to better understand this phenomenon.

The current study has several strengths related to internal and external validity, including rigorous methodology and sound generalizability [32]. These include adequate sample size (n = 1,479) and number of mortality outcomes (n = 284) for conducting a multivariable hazard analysis, prospective evaluation of direct hard-endpoints (all-cause mortality), a lengthy follow up time (8.9±4.5 years) as well as variety of statistical analyses (categorical, continuous, survival, sensitivity). The risk models were adjusted for established, health-related confounders to extract the independent association between dyslipidemia markers and mortality, and a number of sensitivity analyses addressed potential biases and confounding. Mortality outcomes were verified through the Veterans Affairs computerized medical records system, which has been demonstrated to be comparatively accurate and complete [22]. Additional strengths include utilizing established criteria for dyslipidemia and providing clinical and practical perspectives on risk [3]. The study also has several limitations. First, although risk models were adjusted for established confounders, similar to previous studies [7–19], data on dietary habits were not available, which may have had an influence on risk. Second, consistent with previous reports [7–19], analyses were conducted on cholesterol levels only; data on triglycerides and other biomarkers were not available. Third, Veteran participants are a unique population with a rich mixture of co-morbidities which may have influenced the results by selection bias. However, participant characteristics were in alignment with the general US population [33], and the findings were consistent with previous reports [7–19], providing reasonable confidence for generalization. Fourth, the study was limited to men, and the extent to which the findings apply to women requires further investigation. Finally, the findings demonstrated a significant association between exposure (dyslipidemia markers) and an outcome (all-cause mortality) but preclude ascertaining a cause-and-effect relationship, as well as overtime change in cholesterol and initiating medications were not accounted for.

## Conclusions

In utilizing clinically established criteria for dyslipidemia [3], the current study demonstrated that higher TC and LDL-C levels were paradoxically and independently associated with lower risk of all-cause mortality and better survival in men. These findings suggest the need for rigorous reevaluation of dyslipidemia risk and the utility of cholesterol lowering medications. Comprehensive systematic reviews and meta-analyses are needed to shed additional light on this topic.

## Supporting information

S1 File Data of statistical analysis. (PDF)
