# Supplementary material for: Dyslipidemia paradox: Analysis from the veterans exercise testing study
Source: PLoS One. 2023 Jul 19;18(7):e0287923. doi: 10.1371/journal.pone.0287923 (PMC10355403; doi:10.1371/journal.pone.0287923)
Supplement: S1 File — (PDF) [file pone.0287923.s001.pdf]

Data of Statistical Analysis

Categorical Variable Codings[a]

		Frequency	(1)	(2)
LDC_C_130_190[b]	1.00=<130	501	0	0
	2.00=130-189.9	650	1	0
	3.00==>190	278	0	1

a. Category variable: LDC_C_130_190 (LDC_C_130_190)

b. Indicator Parameter Coding

Omnibus Tests of Model Coefficients[a]

-2 Log Likelihood	Overall (score)			Change From Previous Step			Change From Previous Block		
	Chi-square	df	Sig.	Chi-square	df	Sig.	Chi-square	df	Sig.
3533.169	214.295	12	<.001	215.638	12	<.001	215.638	12	<.001

a. Beginning Block Number 1. Method = Enter

Variables in the Equation

	B	SE	Wald	df	Sig.	Exp(B)	95.0% CI for Exp(B)	
							Lower	Upper
LDC_C_130_190			6.227	2	.044			
LDC_C_130_190(1)	-.296	.137	4.652	1	.031	.743	.568	.973
LDC_C_130_190(2)	-.380	.184	4.273	1	.039	.684	.477	.980
risk_factors__hypertension	-.032	.136	.057	1	.812	.968	.741	1.265
risk_factors__family_cad	.002	.152	.000	1	.989	1.002	.744	1.350
risk_factors__alcohol	-.353	.365	.939	1	.333	.702	.344	1.435
meds__antihypertensive	.422	.146	8.304	1	.004	1.524	1.144	2.030
meds__statins	-1.087	.210	26.716	1	<.001	.337	.223	.509
CVD_present	.616	.129	22.774	1	<.001	1.851	1.437	2.383
Age_new_column	.062	.007	81.644	1	<.001	1.064	1.050	1.078
Obesity_Risk_Factor	.090	.132	.467	1	.494	1.094	.845	1.418
Diabetes_New	.229	.148	2.395	1	.122	1.257	.941	1.679
risk_factors__smoke_id	.569	.094	36.701	1	<.001	1.767	1.469	2.124

Categorical Variable Codings[a]

		Frequency	(1)	(2)
TC_200_240[b]	1.00=<200	762	0	0
	2.00=200-239.9	409	1	0
	3.00==>240	258	0	1

a. Category variable: TC_200_240 (TC_200_240)

b. Indicator Parameter Coding

Omnibus Tests of Model Coefficients[a]

-2 Log Likelihood	Overall (score)			Change From Previous Step			Change From Previous Block		
	Chi-square	df	Sig.	Chi-square	df	Sig.	Chi-square	df	Sig.
3532.762	213.658	12	<.001	216.045	12	<.001	216.045	12	<.001

a. Beginning Block Number 1. Method = Enter

Variables in the Equation

	B	SE	Wald	df	Sig.	Exp(B)	95.0% CI for Exp(B) Lower	Upper
TC_200_240			6.065	2	.048			
TC_200_240(1)	-.145	.141	1.045	1	.307	.865	.656	1.142
TC_200_240(2)	-.457	.188	5.902	1	.015	.633	.438	.915
risk_factors__hypertension	-.052	.137	.144	1	.704	.949	.726	1.241
risk_factors__family_cad	.000	.152	.000	1	1.000	1.000	.742	1.347
risk_factors__alcohol	-.342	.364	.884	1	.347	.710	.348	1.450
meds__antihypertensive	.412	.146	7.911	1	.005	1.509	1.133	2.011
meds__statins	-1.044	.208	25.109	1	<.001	.352	.234	.530
CVD_present	.634	.128	24.428	1	<.001	1.885	1.466	2.423
Age_new_column	.062	.007	80.764	1	<.001	1.064	1.049	1.078
Obesity_Risk_Factor	.089	.132	.454	1	.500	1.093	.844	1.416
Diabetes_New	.210	.148	2.002	1	.157	1.233	.922	1.650
risk_factors__smoke_id	.568	.094	36.528	1	<.001	1.765	1.468	2.122

Case Processing Summary

LDC_C_130_190	Total N	N of Events	Censored	
			N	Percent
<130	521	115	406	77.9%
130-189.9	675	123	552	81.8%
=>190	283	46	237	83.7%
Overall	1479	284	1195	80.8%

Means and Medians for Survival Time

LDC_C_130_190	Mean[a]				Median			
	Estimate	Std. Error	95% Confidence Interval		Estimate	Std. Error	95% Confidence Interval	
			Lower Bound	Upper Bound			Lower Bound	Upper Bound
<130	14.087	.319	13.462	14.712	.	.	.	.
130-189.9	15.322	.238	14.855	15.789	.	.	.	.
=>190	16.035	.327	15.395	16.675	.	.	.	.
Overall	15.262	.169	14.931	15.592	.	.	.	.

a. Estimation is limited to the largest survival time if it is censored.

Overall Comparisons

	Chi-Square	df	Sig.
Log Rank (Mantel-Cox)	13.322	2	.001

Test of equality of survival distributions for the different levels of LDC_C_130_190.

Case Processing Summary

TC_200_240	Total N	N of Events	Censored	
			N	Percent
<200	794	171	623	78.5%
200-239.9	420	76	344	81.9%
=>240	265	37	228	86.0%
Overall	1479	284	1195	80.8%

Means and Medians for Survival Time

TC_200_240	Mean[a]				Median			
			95% Confidence Interval				95% Confidence Interval	
	Estimate	Std. Error	Lower Bound	Upper Bound	Estimate	Std. Error	Lower Bound	Upper Bound
<200	14.636	.245	14.157	15.116	.	.	.	.
200-239.9	15.464	.308	14.861	16.068	.	.	.	.
=>240	16.377	.314	15.762	16.991	.	.	.	.
Overall	15.262	.169	14.931	15.592	.	.	.	.

a. Estimation is limited to the largest survival time if it is censored.

Overall Comparisons

	Chi-Square	df	Sig.
Log Rank (Mantel-Cox)	14.546	2	<.001

Test of equality of survival distributions for the different levels of TC_200_240.
